# Synovial Cyst: A Culprit for Recalcitrant Iliotibial Band Syndrome: A Case Report

**DOI:** 10.5704/MOJ.1511.011

**Published:** 2015-11

**Authors:** CSN Yeoh, GHJ Lim, SS Sathappan

**Affiliations:** Department of Othopaedic Surgery, Tan Tock Seng Hospital, Singapore

**Keywords:** Knee, Iliotibial band syndrome, Synovial, Cyst

## Abstract

We present the case of a 56-year old gentleman who presented with recalcitrant iliotibial band (ITB) friction syndrome which did not improve with various modalities of conservative treatment. Magnetic Resonance Imaging (MRI) of the affected knee did not show pathology typical of ITB friction syndrome. However, open exploration revealed a synovial cyst deep to the iliotibial band, abutting against the anterolateral capsule. The presence of distinctive clinical signs on physical examination should alert clinicians to consider knee synovial cyst as a differential diagnosis when dealing with recalcitrant ITB syndrome.

## Introduction

Iliotibial band syndrome is a common cause of lateral knee pain, especially in active athletes. It is postulated to arise from repetitive friction of the iliotibial band over the lateral femoral condyle during flexion and extension of the knee, leading to inflammation and pain^1^. Other common differential diagnoses include lateral meniscal tear, lateral compartmental arthropathy, biceps femoris tendinopathy, patello-femoral pain syndrome and lateral collateral ligament pathology. However, rare causes such as a knee synovial cyst, can also present as iliotibial band syndrome, as first described by Costa *et al* in 2004^2^. We are reporting the second documented case.

## Case Report

A 56-year old gentleman was initially seen in our clinic for right knee pain and effusion with limitation of range of motion (10-110 degrees). MRI showed a 7mm osteochondral defect over the femoral sulcus (Figure 1A) for which he underwent arthroscopic debridement and TruFit osteochondral grafting. At one month post-operative, he was recovering well with decreasing pain in the right knee with improvement of range of motion (0-120 degrees). However, he reported of a 2-month history of new onset localized pain over the lateral aspect of the ipsilateral knee. The pain was worse when using the stairs.

**Fig. 1a:**
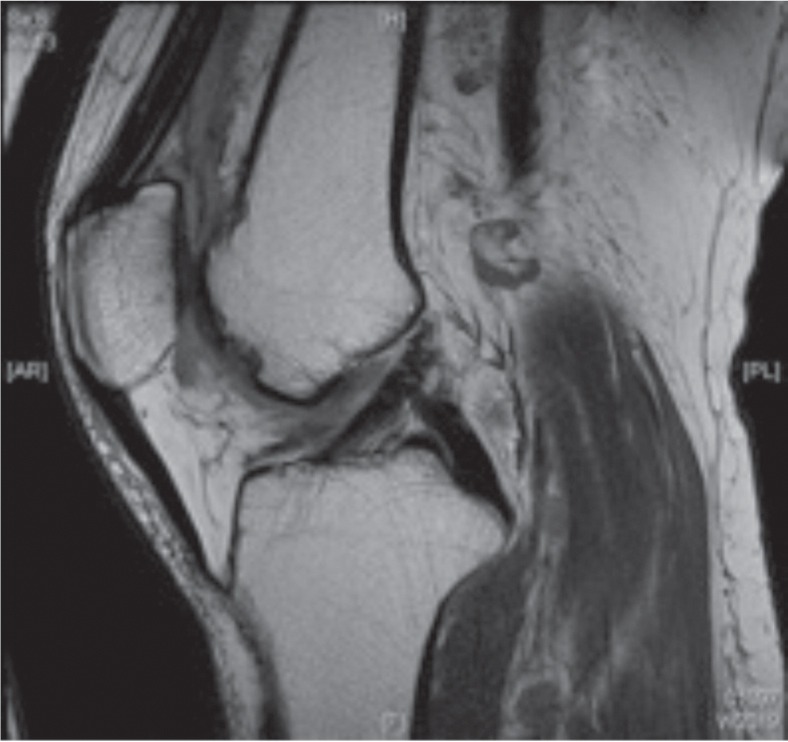
Osteochondral defect over femoral sulcus on MRI.

He was diagnosed with iliotibial band (ITB) syndrome due to the localized pain over the ITB over the lateral joint line. A 4-month course of conservative management with exercises and corticosteroid injection did not improve his symptoms. Repeat MRI of the knee (Figure 1B) performed after failed conservative management did not reveal any iliotibial band pathology or intra-articular pathology that might have contributed to his lateral knee pain.

**Fig. 1b:**
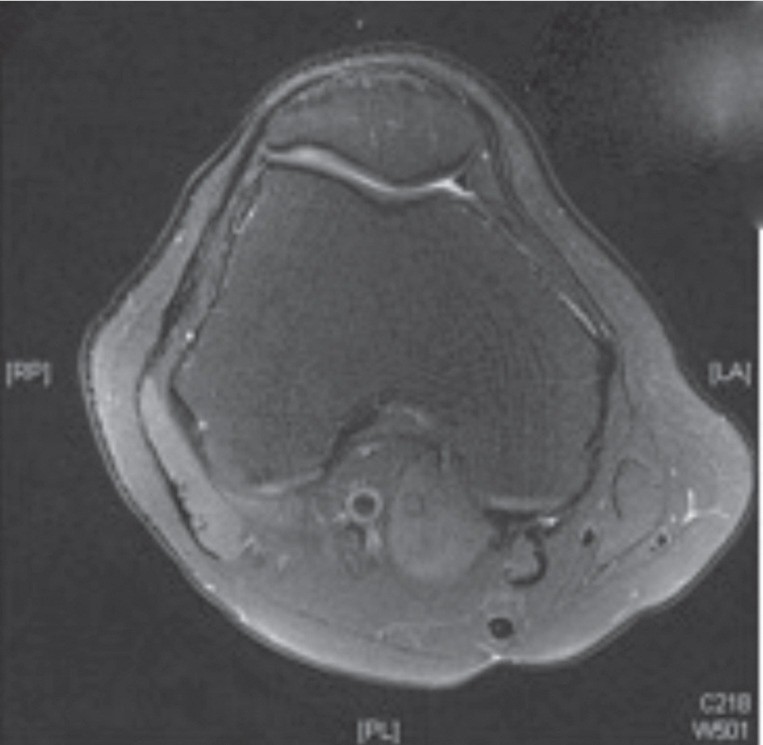
Axial cut of the MRI did not reveal the cyst or other abnormal signal over the iliotibial band.

**Fig. 2:**
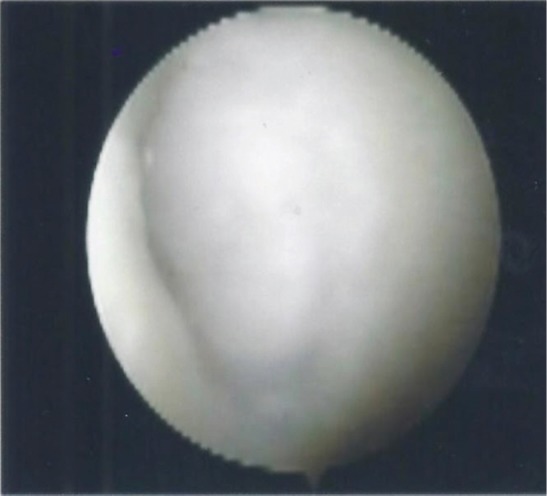
Arthroscopy finding of healed osteochondral defect after grafting.

**Fig. 3:**
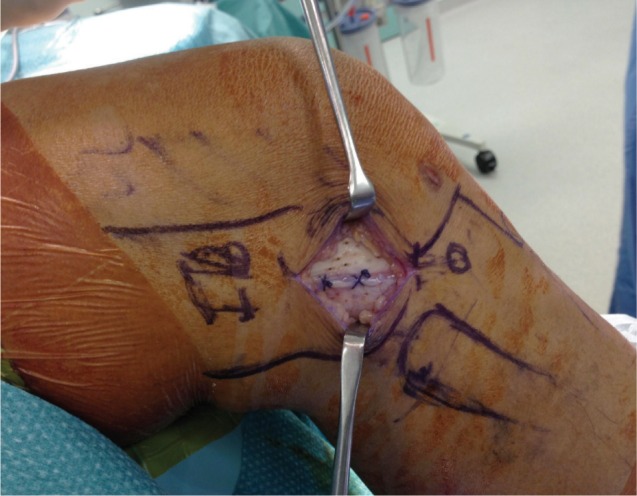
Excision of cyst through longitudinal incision over the ITB and radiofrequency coblation performed after repair of the ITB.

The patient was offered arthroscopic assessment of the knee joint and iliotibial band release. Pre-induction examination in the operating theatre showed that there was localised tenderness over the lateral joint line, beneath the iliotibial band. A vague swelling was palpable with the knee in flexion but the swelling disappeared and pain intensity was markedly reduced on full extension of the knee. Pain was also elicited upon ranging the knee. Then, the area of swelling with maximal tenderness was marked. Arthroscopy of the knee confirmed no intra-articular pathology that could be attributed to the lateral knee pain and the previous osteochondral lesion was found to have healed well with fibrocartilaginous tissue (Figure 2). Exploration of the iliotibial band was then carried out. A 1.3 x 0.9 x 0.4cm cyst was found located deep to the iliotibial band abutting against the antero-lateral capsule. There was pseudo-bursal tissue surrounding the cyst. The cyst was excised followed by radiofrequency coblation of the iliotibial band (Figure 3).

At the first follow up clinic, patient had complete resolution of symptoms. Histology showed a fibro-adipose tissue focally lined with synovial cells and a nodular focus comprising reactive fibroblasts. There was no recurrence of knee pain after one year.

## Discussion

Knee synovial cyst is a rare cause of lateral knee pain, mimicking iliotibial band syndrome. To date, there are only two cases, including ours reported in the literature. The exact pathogenesis of such a condition is unclear. The localized lateral knee pain is likely to arise from the abutment of the cyst against the joint capsule during movement of the iliotibial band.

There is consistency in the clinical findings when comparing our case to the case reported by Costa *et al.* The swelling is more prominent to palpation with flexion of the knee and it disappears on full extension of the knee. Furthermore, the intensity of the pain tends to decrease when the area is palpated with the knee in full extension as demonstrated in our case. These interesting signs are likely due to protection of the cyst from direct compression onto the joint capsule by the tough structure and tension of the iliotibial band.

Pre-operative MRI did not reveal any cyst beneath the iliotibial band as the scan was performed with the knee in extension. There was also no high intensity signal over lateral femoral epicondyle deep to iliotibial band or thickening of the distal iliotibial band that might be seen in classic iliotibial band syndrome^3,4^.

In conclusion, clinicians should consider knee synovial cyst as a differential diagnosis in patients presenting with recalcitrant iliotibial band syndrome, especially those who elicit the distinctive clinical signs described in the report.
